# Comparison of physiological responses of running on a nonmotorized and conventional motor-propelled treadmill at similar intensities

**DOI:** 10.1038/s41598-022-13741-w

**Published:** 2022-07-08

**Authors:** Filipe A. B. Sousa, Fúlvia B. Manchado-Gobatto, Natália de A. Rodrigues, Gustavo G. de Araujo, Claudio A. Gobatto

**Affiliations:** 1 School of Applied Sciences, University of Campinas, Limeira, São Paulo Brazil; 2 Laboratory of Applied Sports Sciences, Federal University of Alagoas, Maceió, Alagoas Brazil; 3 Postgraduate Nutrition Program-PPGNUT, Faculty of Nutrition, Federal University of Alagoas, Maceió, Alagoas Brazil; 4 Faculty of Physical Education, University of Campinas, Campinas, São Paulo Brazil

**Keywords:** Homeostasis, Metabolism, Physiology, Respiration, Rehabilitation

## Abstract

This study aimed to test the agreement of the incremental test's physiological responses between tethered running on a nonmotorized treadmill (NMT) to matched relative intensities while running on a conventional motorized treadmill (MT). Using a within-subject crossover design, nine male recreational runners (age = 22 ± 5 years; height = 175 ± 6 cm; weight = 68.0 ± 16.6 kg) underwent two test sessions: one was an incremental intensity protocol on an MT; the other was on an instrumented NMT. Intensity thresholds at ${\dot{\text V}}$O_2max_, respiratory compensation point (iRCP), and lactate threshold (iLT) were registered for analysis, together with ${\dot{\text V}}$˙O_2_, ${\dot{\text V}}$˙E, ƒ_R,_ and blood lactate concentration ([Lac]). Comparisons were based on hypothesis testing (Student's T-test), effect sizes (Cohen's d), ICC, and Bland Altman analysis. Statistical significance was accepted at p < 0.05. Attained ${\dot{\text V}}$O_2max_ (MT = 52.2 ± 7.3 mL·kg^-1^·min^-1^ vs NMT = 50.1 ± 8.1 mL·kg^-1^·min^-1^) and ${\dot{\text V}}$˙O_2_ at iRCP (MT = 46.3 ± 7.2 mL·kg^-1^·min^-1^ vs NMT = 42.8 ± 9.3 mL·kg^-1^·min^-1^) were not different between ergometers (p = 0.15 and 0.13, respectively), with significant ICCs (0.84 and 0.70, respectively) and Pearson’s correlations (r = 0.87 and 0.76, respectively). The [Lac] at iLT presented poor agreement between conditions. Significant correlations were found (r between 0.72 and 0.83) for relative power values of i${\dot{\text V}}$O_2max_ (6.56 ± 1.28 W·kg^−1^), iRCP (4.38 ± 1.50 W·kg^−1^), and iLT (4.15 ± 1.29 W·kg^−1^) related to their counterpart obtained on MT. Results show that running on an NMT offers a higher glycolytic demand under the same relative internal load as running on an MT but with a similar aerobic response and correlated intensity determination.

## Introduction

Treadmills have been used for training, rehabilitation, and research protocols whenever there is a need to emulate overground running. The use of treadmills has obvious purposes—to control for space, environmental, and load conditions while enabling the attachment of fixed measurement devices^1,2^. Instrumented treadmills have been employed to enhance locomotion gait analysis, allowing the measurement of kinetic parameters with adequate accuracy^1,3^. Considering the growing presence of strength training in long-distance preparation^4^, explained by the benefits of neuromuscular gains on endurance performance and high-intensity running performance^5^, it is expected to see a rising interest in using instrumented treadmills in training and testing routines.

Similar to instrumented treadmills, there is increased attention to nonmotorized treadmills (NMT), because they allow for instantaneous variations in running speed and acceleration^6–8^. On the other hand, the motorized treadmills (MT) are helpful for constant work-rate efforts and an accurate increase in intensity. During long-duration running, acceleration and decelerations are performed at will, with variations caused by course profile, pacing strategy, and competition^9^. The possibility of fast variation on running speed enhances the potential of NMT for the study of training and simulation of performance, a trait that has been associated to an enhanced similarity on kinematics during accelerated running events^3^.

Both MT and NMT can present arguably comparable kinematics to overground running^3,10^, despite differences in kinetics for accelerated events^6,11^. From the physiological, perceptual, and performance standpoints, a recent meta-analysis has shown that the differences between running overground and treadmills are dependent on the running speed assessed^2^. For example, at maximum running intensity, oxygen consumption (${\dot{\text V}}$O_2_) and heart rate (HR) on MT were comparable to overground running^2^. However, at near maximum running (${\dot{\text V}}$O_2_ > 80%), ${\dot{\text V}}$˙O_2_ and blood lactate concentration ([Lac]) were lower for MT than overground.

Among the 34 studies assessed in the Miller et al.^2^ meta-analysis, only three involved NMT, and all using a curved surface, reinforcing the need for further investigation on this topic. At maximal running on NMT, ${\dot{\text V}}$˙O_2_ and HR were similar to overground running, but values of [Lac] and RPE were higher^12^. However, NMT running at matched submaximal speeds showed higher ${\dot{\text V}}$˙O_2_, [Lac], HR, and perception of effort than overground running^7,13^. So, despite similarities to overground running at maximum effort, NMT and MT running present different physiological responses regarding submaximal work-rates, being usually higher for NMT and lower for MT^14^. Recently, it has been shown the need for a 6 to 8% inclination on an MT when running at 10 kmꞏh^−1^ to achieve comparable physiological and perceptual responses to a curved model of NMT^15^. Considering uphill running at MT to present a higher neuromuscular demand compared to level running^16^, this may be the case when comparing MT level running to NMT, thus explaining the higher physiological demands at a same absolute speed.

Correlations between physiological responses after running at maximum effort overground compared with both NMT and MT suggest their usefulness as part of training and testing programs^2,12^. For their use as training devices, the choice between MT and NMT could be driven by the need for a constant work-rate or rapid changes at will in running speed. However, considering the differences in physiological responses at absolute submaximal speeds^14,15^, it is important to confirm if NMT and MT running are physiologically comparable at matched relative workloads. If this premise proves to be true, NMT and MT running at the intensity of a same metabolic threshold should result in similar adaptations to training, expanding the interchangeably use of NMT and MT during a training program.

This study tested the hypothesis of similar oxygen consumption, ventilation, respiratory frequency, and blood lactate concentration at the intensities associated with the lactate threshold, the respiratory compensation point, and the maximum oxygen consumption intensities for both NMT and MT ergometers. If confirmed, the adjustment of submaximal running physiological demands during NMT and MT would be possible. The current study aimed to test the agreement of the physiological responses from an incremental intensity test performed at tethered running on an NMT to their matched relative intensities on an MT.

## Materials and methods

### Participants

Nine male recreational runners (age = 22 ± 5 years; height = 175 ± 6 cm; weight = 68.0 ± 16.6 kg; body fat = 7.2 ± 3.8%; training frequency 3/week, minimum 15 km/week) gave written consent to take part in this investigation, being informed of the benefits and risks of the investigation prior to signing. All procedures were previously approved by the Research Ethics Committee of the School of Medical Sciences (number 28442314.0.0000.5404) and complied with the Declaration of Helsinki.

### Design and procedures

**Figure 1:**
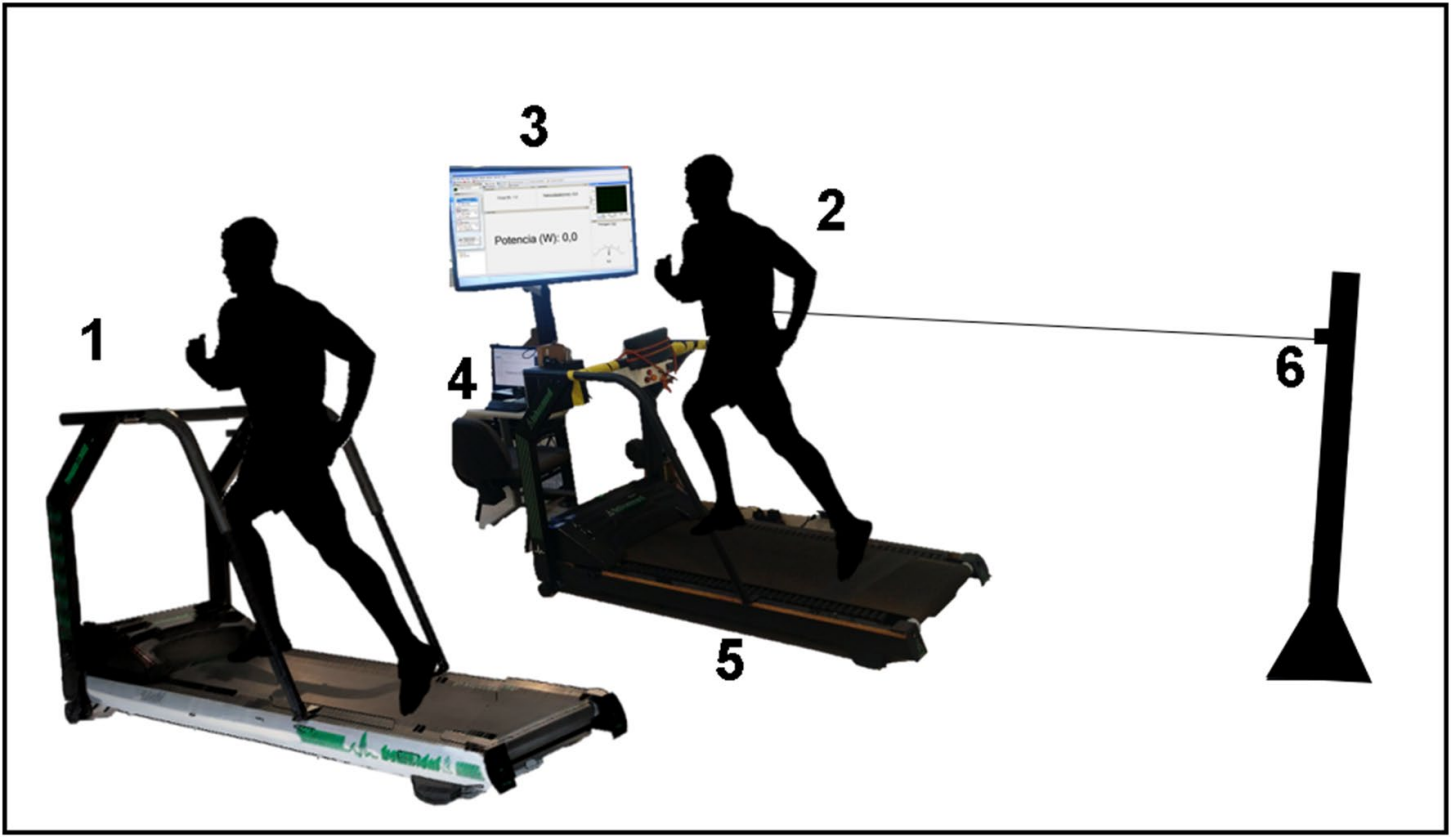
Set-up image for the studied ergometers. (1) conventional MT running; (2) tethered NMT running; (3) monitor for visual feedback of horizontal plane power performance; (4) computer and signal acquisition equipment; (5) instrumented NMT for vertical force measurement; (6) load cell attached to a fixed metal pole enabling heigh adjustment to measure force in the horizontal plane. In both cases, the runners were equipped with a portable gas analysis device at all times.

Previously to the experimental sessions, volunteers visited the laboratory to learn about the protocol and give informed consent for participation. The first visit consisted of anthropometric measures and ergometer familiarization. Then, with a randomized crossover design, volunteers were called back and underwent two test sessions set apart by two to seven days. These two sessions consisted of a warm-up followed by one incremental test performed on either an NMT (custom build from an ATL, Inbrasport®, Brazil^17,18^) or MT (Super ATL, Inbrasport, Brazil) (Fig. 1). The volunteers ran equipped with a portable gas analyzer (K4b^2^, COSMED®, Italy) at all times during MT and NMT testing. Blood samples were taken by the ear lobe at the end of each stage of the incremental intensity test and stored into microtubes (Eppendorf®, 1.5 ml) containing 50 μl of 1% sodium fluoride (NaF) for later analysis (YSI-2300 STAT PLUS, Yellow Springs®, USA).

### Measures

Incremental tests were performed at 0% inclination and had three-minute stages followed by 30-s pauses for blood sample collection. For the MT, the test started at 7 km·h^−1^ with 1 km·h^−1^ increments. Considering absolute speeds do not present similar physiological demands between MT and NMT^7,13^, the work-rate on the NMT was determined by power in the horizontal plane, starting at 80 W with 20 W increments. On the NMT, the intensity was held constant by visual and verbal feedback of horizontal plane power performance. The protocol design was determined by pilot testing with runners of similar characteristics to enable at least five stages for every volunteer. Tests were continued until either exhaustion or the attainment of two of the following criteria: (i) a plateau in ${\dot{\text V}}$O_2_; (ii) R higher than 1.0; (iii) blood lactate concentration higher than 8 mmol·L^−1^. The same criteria were used to determine ${\dot{\text V}}$˙O_2max_. Criteria for the plateau in ${\dot{\text V}}$O_2_ was defined based on individual variability of ${\dot{\text V}}$˙O_2_ in respect to the work-rate, i.e. when the difference between stages was less than half of the expected increase in ${\dot{\text V}}$O_2_ obtained from the submaximal work-rate stages regression analysis^19^.

**Figure 2:**
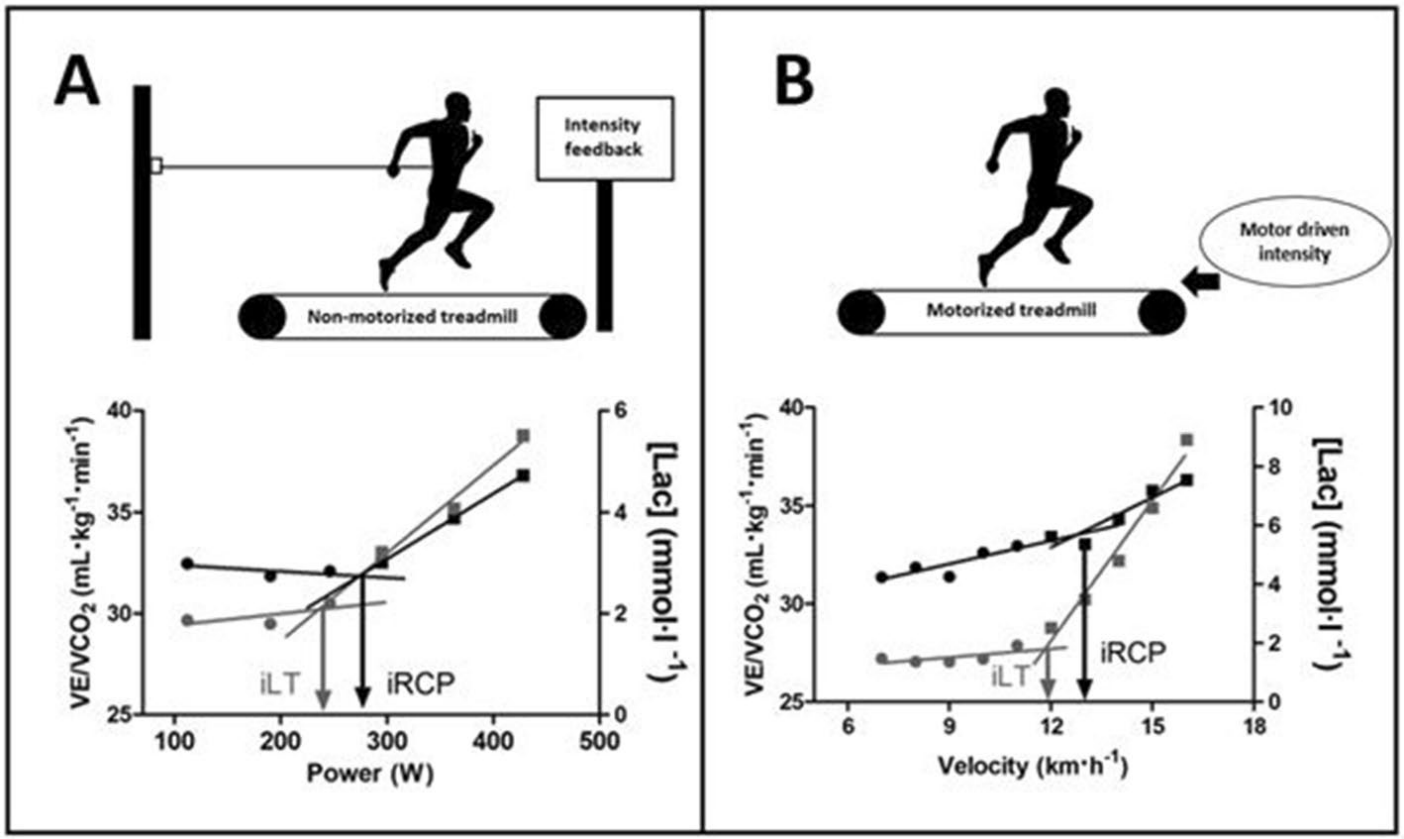
Example of the nonmotorized treadmill (**A**) and motorized treadmill (**B**) set up to determine iRCP and iLT; power is expressed as the resultant of horizontal and vertical orientations. Arrows are merely indicative. *[Lac]* blood lactate concentration, ${\dot{\text V}}$E/${\dot{\text V}}$˙CO_2_ quotient between ventilation and CO_2_ production.

${\dot{\text V}}$˙O_2_, carbon dioxide output (${\dot{\text V}}$CO_2_), minute pulmonary ventilation (${\dot{\text V}}$E), and respiratory frequency (ƒ_R_) at a given stage was the average value of the last 30 s of data. The first intensity to elicit ${\dot{\text V}}$˙O_2max_ was defined as i${\dot{\text V}}$˙O_2max_. At the i${\dot{\text V}}$˙O_2max_ stage, both ƒ_R_ and ${\dot{\text V}}$E were retained for analysis. The ${\dot{\text V}}$O_2_ at the respiratory compensation point (RCP) and its intensity of occurrence (iRCP) were determined by bi-segmented analysis of ${\dot{\text V}}$E/${\dot{\text V}}$CO_2_^20^. Similarly, the lactate threshold (LT) and its respective intensity (iLT) were determined by the analysis of the blood lactate concentration and running work-rate^21^(Fig. 2). Regression analyses were performed to find the intersection point for a given parameter′ s first and second linear regression^22^. The ${\dot{\text V}}$O_2_ at iRCP was also normalized to ${\dot{\text V}}$O_2max_. iRCP and iLT were expressed as their respective intensities, as well as normalized for i${\dot{\text V}}$O_2max_ for paired comparison between conditions.

The NMT ergometer was adapted from a commercial model (Super ATL, Inbrasport®, Brazil) as detailed elsewhere^17,18^ has a running surface of 196 × 64 cm. Volunteers ran with a load cell in series with an inextensible steel cable attached to their waists. The absence of a driving motor enables the runner to perform the propelling force needed to run during the entire test. The NMT was upgraded to register vertical forces, mounted on four load cells to register the force in the vertical direction. After test completion, the resultant force was calculated using the vertical and horizontal plane force measurements, and mechanical power was obtained as the product between velocity and resultant force. The signal acquisition system (DAQ module, amplifier, and Hall effect sensor) was set to record data at 1000 Hz. The force sensors were calibrated using known weights before each test. Force signals were filtered using a low-pass, fourth-order Butterworth filter, with a cutoff frequency of 10 Hz, to account for noise from the electrical grid (50–60 Hz) and natural vibrations (> 15 Hz).

### Statistical analyses

Data were described as mean and SD. After data normality confirmation (Lilliefors test), paired Student's t-tests were used to verify significant differences in physiological parameters between MT and NMT. Pearson's correlation coefficient was adopted to verify consistency. The sample size was defined by convenience but considered the possible statistical power analysis. Using G*Power^23^, a t-test for dependent means (ES > 0.8 and α > 0.5) and a point biserial model correlation (ρ^2^ > 0.5 and α > 0.5) returned statistical power = 0.7 for a sample size of nine. Cohen's d was calculated to access effect sizes (ES), being d ≤ 0.20 a small effect, d ≤ 0.50 a medium effect, and d ≤ 0.80 a large effect. For concordance level between tests, ICC for absolute agreement (3,1) was performed, together with bias (MD) and Limits of Agreement (LA) from Bland Altman's analysis. For ICC, thresholds were 0.99, 0.90, 0.75, 0.50, and 0.20, for extremely-high, very-high, high, moderate, and low^24^. Both concordance tests were performed using a custom Matlab function (MATLAB 6.0, MathWorks Inc.®), while the remaining statistical analysis used Statistica 7.0 (StatSoft®). Statistical significance was accepted at p < 0.05.

## Results

**Figure 3:**
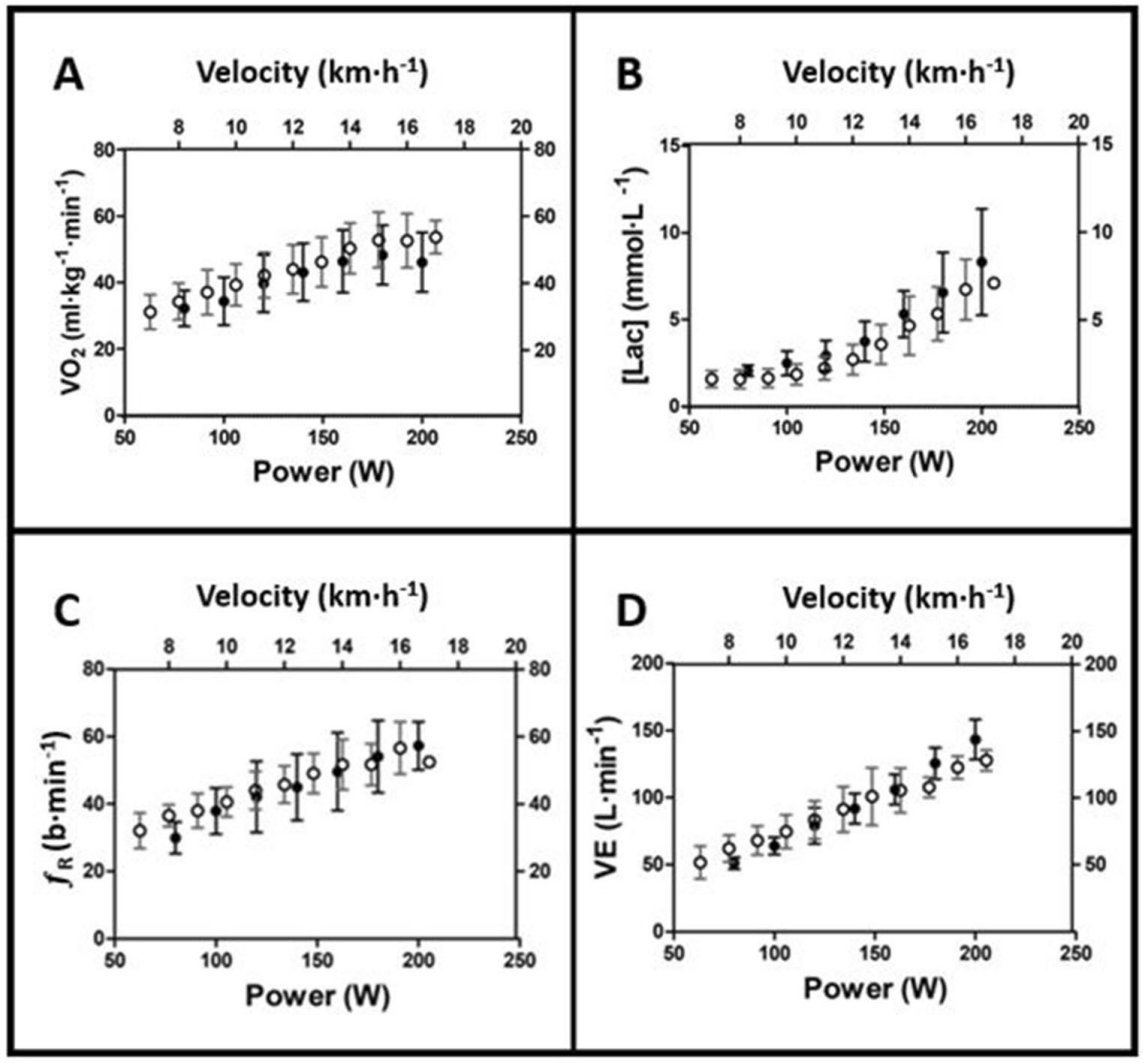
Descriptive data for ${\dot{\text V}}$O_2_ (**A**), blood lactate concentration (**B**), ƒ_R_ (**C**), and ${\dot{\text V}}$E (**D**) for the incremental intensity test performed on NMT (filled circles) and MT (open circles). Power data in NMT is expressed considering only the horizontal orientation, to maintain the same intensity for each subject (see “Methods” section for detail).

Descriptive data for ${\dot{\text V}}$O_2_ and blood lactate concentration for both incremental tests are depicted in Fig. 3. It is possible to see a linear increase in ${\dot{\text V}}$˙O_2_ related to exercise intensity and a plateau for the last stages of incremental tests performed on both NMT and MT. Additionally, blood lactate concentration showed higher values for NMT than MT, with wider SDs. Of the nine runners, six presented a ${\dot{\text V}}$˙O_2_ plateau at the end of the test for MT and five for NMT. All runners presented a ${\dot{\text V}}$O_2_ plateau under at least one studied condition. For the remaining tests, ${\dot{\text V}}$˙O_2max_ was confirmed by the meeting of the other two criteria.

**Figure 4:**
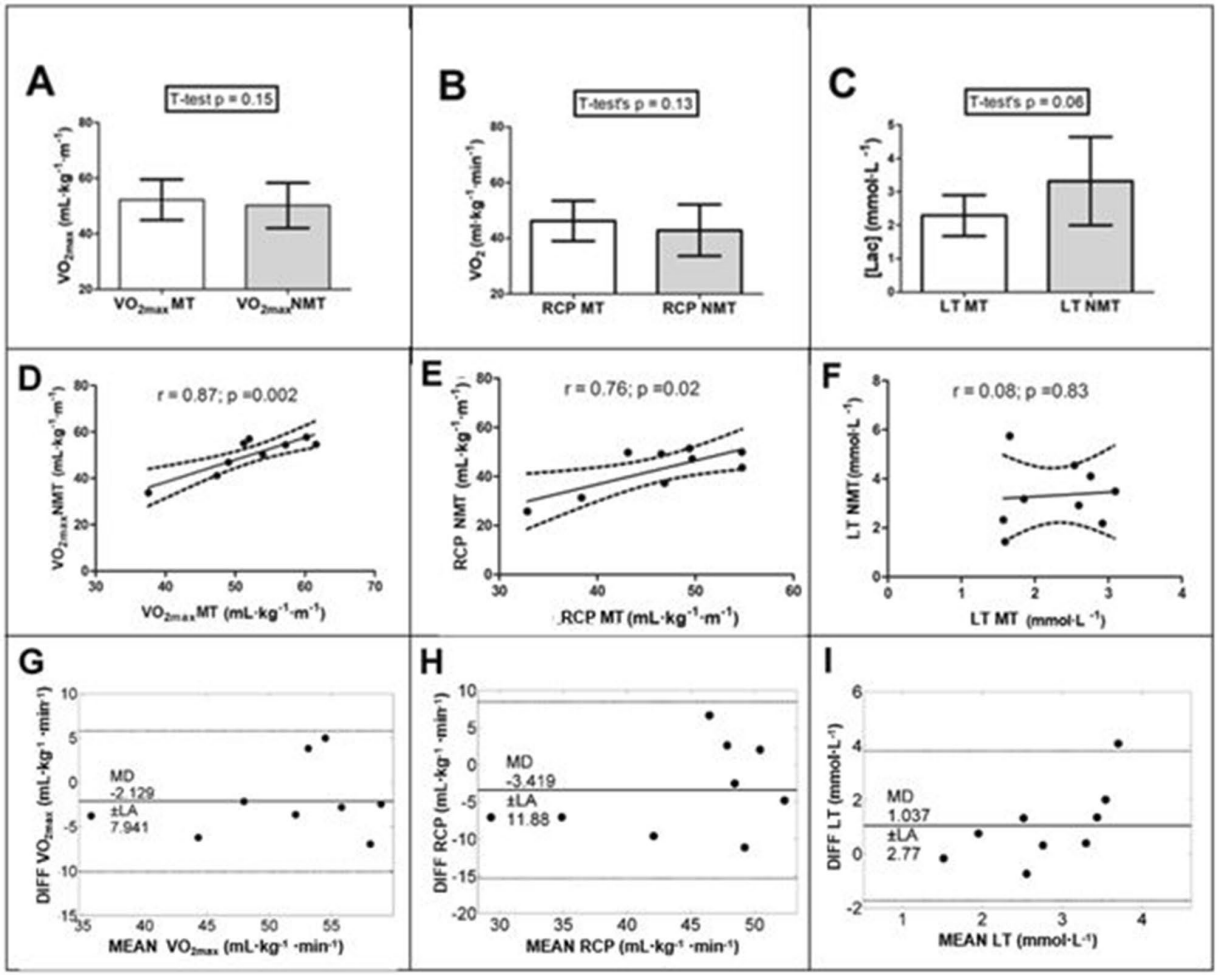
Comparison of ${\dot{\text V}}$O_2max_ (**A**), ${\dot{\text V}}$O_2_ in iRCP (**B**) and [Lac] values in iLT (**C**) between the incremental tests in different ergometers, with their respective correlation (**D–F**) and Bland Altman plots (**G–I**); *[Lac]* blood lactate concentration, *iRCP *second metabolic threshold intensity determined by respiratory parameters, *iLT *second metabolic threshold intensity determined by lactate; ${\dot{V}}$*O*_*2max*_ maximal oxygen consumption, *MT* motorized treadmill, *NMT* nonmotorized treadmill.

Attained ${\dot{\text V}}$˙O_2max_ (MT = 52.2 ± 7.3 mL·kg^−1^·min^−1^ vs NMT = 50.1 ± 8.1 mL·kg^−1^·min^−1^) did not present significant difference between tests (Fig. 4A), with ES = 0.27. Correlation between them was also high and significant (Fig. 4D). High ICC (0.84; p < 0.001) confirmed agreement between ${\dot{\text V}}$˙O_2max_ in the two incremental tests, with only little bias presented by Bland Alan plots (Fig. 4G). Additionally, no significant difference was registered between ergometers (p = 0.54 and 0.07, respectively; ES = 0.28 and 0.68) for values of ƒ_R_ (MT = 55 ± 5 b·min^−1^ and NMT = 57 ± 9 b·min^−1^) and ${\dot{\text V}}$E (MT = 121.1 ± 14.7 L·min^−1^ and NMT = 131.9 ± 16.8 L·min^−1^) at i${\dot{\text V}}$˙O_2max_. However, ƒ_R_ and ${\dot{\text V}}$E did not present significant Pearson’s correlation between conditions (r = 0.21; p = 0.59 and r = 0.55; p = 0.13, respectively) and had low ICCs (ICC = 0.18; p = 0.31 and ICC = 0.46 p = 0.06, respectively). Additionally, Bland Altman analysis for ƒ_R_ and ${\dot{\text V}}$E at i${\dot{\text V}}$O_2max_ reinforced an inconsistency between conditions, based on high Limits of Agreement for ƒ_R_ (MD = −1.75 and LA = 19.25 b·min^−1^) and ${\dot{\text V}}$E (MD = −10.81 and LA = 29.64 L·min^−1^).

The ${\dot{\text V}}$O_2_ at iRCP (MT = 46.3 ± 7.2 mL·kg^−1^·min^−1^ vs. NMT = 42.8 ± 9.3 mL·kg^−1^·min^−1^) was not statistically different between tests (Fig. 4B), with ES = 0.42, and there was a significant Pearson correlation coefficient between the two measures (Fig. 4E). Also, ${\dot{\text V}}$˙O_2_ at iRCP presented moderate but significant ICC (ICC = 0.70; p = 0.008) and low bias (Fig. 4H) between the two ergometers. Lactate concentration in iLT (MT = 2.29 ± 0.61 mmol·L^−1^ vs NMT = 3.33 ± 1.32 mmol·L^−1^) presented only a tendency towards significant difference (Fig. 4C), yet with ES being equal to 0.66, an absence of a significant Pearson correlation coefficient (Fig. 4F), low ICC (ICC = 0.04; p = 0.43) and high bias (Fig. 4–I). No significant correlation was found in the Bland Altman analysis for all parameters, indicating no tendency in bias along the range of observations^25^.

Intensity parameters for the MT incremental test (i${\dot{\text V}}$O_2max_ = 14.7 ± 0.9 km·h^–1^; iRCP = 12.3 ± 1.6 km·h^–1^; iLT = 11.8 ± 1.4 km·h^–1^) were better correlated with the ones obtained on the NMT test when relativized by body mass (i${\dot{\text V}}$O_2max_ = 6.56 ± 1.28 W·kg^–1^; iRCP = 4.38 ± 1.50 W·kg^–1^; iLT = 4.15 ± 1.29 W·kg^–1^) than in absolute units (i${\dot{\text V}}$O_2max_ = 440 ± 107 W; iRCP = 284 ± 81 W; iLT = 271 ± 71 W) (Table 1). Despite correlation, when normalized by i${\dot{\text V}}$O_2max_, iRCP (MT = 83.9 ± 8.3%; NMT = 66.6 ± 17.4%; p = 0.01) and iLT (MT = 80.4 ± 6.9%; NMT = 63.3 ± 14.6%; p < 0.01) presented significantly higher values during the MT test than the NMT. Even then, ${\dot{\text V}}$O_2_ at iRCP normalized by ${\dot{\text V}}$O_2max_ was not different between conditions (MT = 88.5 ± 5.8%; NMT = 84.8 ± 7.0%; p = 0.01).

**Table 1:** Correlations between intensity parameters measured on the nonmotorized treadmill and motorized treadmill, expressed as r(p).

	iRCP (km·h^−1^)	iLT (km·h^−1^)	i${\dot{\text V}}$O_2max_ (km·h^−1^)
iRCP (W)	0.62 (0.08)	0.66 (0.05)	0.50 (0.17)
iRCP (W·kg^−1^)	0.80 (0.01)*	0.81 (0.008)*	0.71 (0.03)*
iLT (W)	0.53 (0.14)	0.62 (0.08)	0.49 (0.18)
iLT (W·kg^−1^)	0.78 (0.01)*	0.83 (0.006)*	0.79 (0.01)*
i${\dot{\text V}}$O_2max_ (W)	0.06 (0.89)	0.07 (0.85)	− 0.08 (0.84)
i${\dot{\text V}}$O_2max_ (W·kg^−1^)	0.73 (0.02)*	0.75 (0.02)*	0.72 (0.03)*

*iRCP* metabolic threshold intensity determined by respiratory parameters, *iLT *metabolic threshold intensity determined by lactate; *i*${\dot{\text V}}$*O*_*2max*_ maximal oxygen consumption intensity.

*p < 0.05.

## Discussion

The current study aimed to compare parameters of an incremental intensity test performed on an NMT and an MT, matched by physiological thresholds. Results indicated similar oxygen consumption at i${\dot{\text V}}$˙O_2max_ and iRCP. The poor agreement between conditions for blood lactate concentration at iLT may be justified by different exertion of force between ergometers. In general, the intensities (iRCP, iLT, and i${\dot{\text V}}$˙O_2max_) presented higher correlation coefficients when power was relativized by body mass.

Comparisons between NMT and MT have been made, often considering absolute work-rates and using a curved NMT. The maximal attained speed and the self-selected submaximal intensities referred to as easy and moderate are always lower in NMT than MT^13,14,26^. For speeds ranging from 5 to 16 km·h^−1^, differences in ${\dot{\text V}}$˙O_2_, heart rate, and rate of perceived exertion were found between NMT and MT running^7,13,14,26^. There were no significant differences between NMT and MT for [Lac] while running at 12 km·h^−1^, while it was higher on the NMT for 14 and 16 km·h^−1^^13^. The present study furthers these results by comparing the two ergometers when running at intensities matched by individual physiological thresholds.

For oxygen consumption at both i${\dot{\text V}}$˙O_2max_ and iRCP, the absence of significant difference, together with lower bias and significant correlation between running conditions, supports the hypothesis of a physiological equivalence in these intensities for the two ergometers. Previous studies have shown oxygen consumption and blood lactate concentration responses to be modality dependent for the same relative intensity, attributing this to differences in the activated muscle mass and body position between cycle and running or swimming and running^27–29^. Despite this, both ergometers used in this study simulated the exercise pattern of running; hence, differences in ${\dot{\text V}}$˙O_2_ for these relative intensities of exercise were not found.

The comparison between ergometers for blood lactate concentration at iLT, however, presented p = 0.06, with no correlation and significant bias between MT and NMT. There is a notion that the runner does not exert as much force in propelling the body while running on an MT as during overground running, decreasing anterior and medial ground reaction forces and presenting changes in electromyographical activity^10,11,30^. The evidence on why does this may happen is conflicting. Among the possible explanations for having less propulsive forces during MT than during overground running are the lack of air drag force, intra-belt fluctuations, differences in surface stiffness, and lack of familiarization – which could result in higher stride frequency and lower push of per step^10^. In constant submaximal work-rate, air resistance and belt fluctuations may be considered low and thus negligible.

Differences in surface stiffness could be present when comparing the NMT and MT used here, considering that despite being from the same manufacturer, the NTM had its running surface adapted to register force. A stiffer running surface presents lower vertical deformation and shock absorption but higher energy restitution^31^. However, this difference in surface stiffness between treadmills is expected to produce differences in oxygen consumption at submaximal work-rates^32^, which was not the case for iRCP in this study.

On the NMT, the force to push the body forward is simulated when the runner himself propels the treadmill belt. Whatever the explanations for it (surface stiffness, belt speed variations, a higher need to push-off, or all), it seems that running on an NMT may require higher muscular forces and recruit less oxidative muscle fibers than on an MT. A higher force exertion could explain the high effect size, poor correlations (Pearson and ICC), and higher bias for blood lactate profile at iLT between the NMT test and the conventional treadmill.

Exercise at iLT is characterized as within the intense exercise domain^33^. At this intensity, it is expected that three phases will represent ${\dot{\text V}}$˙O_2_ kinetices: phase 1 is a delay in pulmonary responses due to O_2_ stores within the muscles and blood; phase 2 is characterized by a rapid increase in ${\dot{\text V}}$˙O_2_ until phase 3, where stabilization of physiological parameters occurs^33^. During phase 3, pulmonary ${\dot{\text V}}$˙O_2_ reflects muscle oxygen usage. However, the exponential rise in ${\dot{\text V}}$O_2_ along phase 2 is supplemented by ATP re-phosphorylation by oxygen-independent pathways, such as the anaerobic phase of glycolysis, which results in blood lactate accumulation.

As previously mentioned, on the NMT, the muscular effort must be higher than on MT due to the absence of a motor-driven treadmill belt or surface stiffness. Training on an NMT is known to enhance concentric strength of the quadriceps in detriment to the hamstrings compared to MT^34^. A higher muscular mass recruitment or even recruitment of more type II muscle fibers^35,36^ may be responsible for increases in blood lactate concentration at a given intensity. The type of fiber recruitment may explain the higher values for this variable when using the NMT than on the MT, as found in our study, and for overground running, as found elsewhere^37–39^. This way, running on an NMT could offer higher stress for the glycolytic pathway of ATP resynthesis, even with similar relative intensities than on the MT. Higher glycolytic demand and recruitment of less efficient fiber types may be corroborated by iRCP and iLT, occurring earlier on the NMT than the MT in relation to i${\dot{\text V}}$O2max. At first, it is possible that there is an overall lower running efficiency when running on the NMT than the MT. However, the relative and absolute aerobic demand is similar at iRCP under both conditions. This way, earlier blood lactate production while running on NMT could explain iRCP lower intensities relative to ${\dot{\text V}}$˙O_2max_.

Furthermore, studies comparing blood lactate concentration at iLT obtained by an incremental test performed on different surfaces have often found lower values on the MT than the on-field testing^37–39^. Furthermore, although the results presented here could not compare running on an NMT with overground, one previous investigation on 5-km performance registered higher post-exercise blood lactate concentration on the NMT than overground, without differences in ${\dot{\text V}}$O_2_^12^. In contrast, submaximal intensities while running on MT elicited lower [Lac] (around 1.26 mmol·L^−1^ less whit 0% grade and 0.52 mmol·L^−1^ with 1%), compared to overground, also without differences in ${\dot{\text V}}$˙O_2_^2^. These results corroborated with our data on similarities of ${\dot{\text V}}$O_2_ at iRCP and the higher [Lac] at LT, which suggest that blood lactate concentration is more sensitive to changes in the running ergometry than ${\dot{\text V}}$˙O_2_.

It should be noted here that ƒ_R_ and ${\dot{\text V}}$˙E were not as consistent between ergometers as ${\dot{\text V}}$˙O_2_ was at the same relative intensity (i${\dot{\text V}}$˙O_2max_). Nicolo and colleagues^40^ recently presented an interesting insight into the myriad of inputs which controls ${\dot{\text V}}$˙E, being both biochemical (e.g., blood lactate concentration) and oxidative demands, together with muscle afferent feedback and central command as the fast inputs to drive the ƒ_R_ response to exercise. Specifically, research showing a strong relationship between the rating of perceived exertion and ƒ_R_ during cycling exercise^41^ supports the possibility of this parameter’s sensitivity to the overall effort. Considering the ergometer comparison scenario presented here, the metabolic demand between conditions is similar from an oxidative standpoint but differs in its glycolytic requirement. Ventilatory parameters such as ${\dot{\text V}}$˙E and ƒ_R_ are, to some extent, are being influenced by both inputs. This way, blood lactate concentration and ${\dot{\text V}}$˙O_2_ may be more specific for glycolytic and oxidative exercise demands, respectively, whereas ${\dot{\text V}}$E and ƒ_R_ may respond to the overall effort being performed.

We used a tethered running set-up and an instrumented NMT to obtain these results and compared them to the physiological demands of running at a conventional MT. The NMT incremental protocol had its work-rate controlled by power rather than speed. Controlling for power was the case, considering that absolute speeds already had been shown to present significant differences between NMT and MT submaximal running^7,13,14,26^. The measurement of power considers speed and exerted force; this latter is expected to be different between ergometers, although this particular experimental design may not prove such an assumption. Nevertheless, the work-rates associated with thresholds were correlated between ergometers. The improvement of the relationship between velocity on MT and power on NMT at i${\dot{\text V}}$˙O_2max_, iRCP, and iLT relative to body mass, may be explained by differences in running efficiency between heavier and lighter runners^29^. Body mass is a known factor that influences running efficiency and the cost of running^42^. Heavier runners are at a disadvantage because of their need to exert disproportionally more force, which is not entirely converted to velocity.

Sirotic and Coutts^43^ performed a team sports simulation using a nonmotorized treadmill (NMT). They justified using an NMT by considering volitional acceleration changes inherent to the type of exercise. The authors categorized efforts relative to maximum sprinting speed, not focusing on physiological alterations dependent on exercise intensity domains. In sports simulations of this nature, the determination of i${\dot{\text V}}$˙O_2max_, iRCP, and iLT on an NMT, as shown here, could help to improve how to categorize relative internal load.

Results presented here indicate the possibility of controlling relative intensity using an incremental test on an NMT with work-rate results related to those from an MT. A possible difference in the glycolytic demand between running on NMT and MT must be considered. Training adaptations of the athlete′s running profile could be monitored using power units, which improve comparison to other ergometers and enable the evaluation of team sports athletes, where force and power may be even more critical for success. Athletes and coaches may use MT and NMT as training options for submaximal and maximal efforts. We suggest relativizing the work-rate for RCP or LT if there is any intent to use those ergometers interchangeably. Even though, at the same relative intensities, NMT running seems to present higher [Lac] than MT running, which should be considered for training aspects.

Among the study’s limitations are the training level of the runners. Previous literature has shown that absolute running speed is a factor to be considered when studying the difference of physiological demands between treadmill and overground running^2,7^. So, it is necessary to investigate if experienced long-distance runners present the same differences in physiological responses as shown for recreational runners at comparable relative intensities. It is known that the fitness level, based on higher anaerobic capacity and higher RCP in relation to ${\dot{\text V}}$˙O_2max,_ can change ${\dot{\text V}}$˙O_2_ plateau occurrence^44^, for example. The sample size is also a limitation to be considered, even if the statistical power able to be achieved in this study was described in the methods section. The report of achieved effect sizes, alpha levels, ICC for absolute agreement, and Bland–Altman analysis intended to enhance the quality of the data compared to other investigations. Future studies may consider comparing NMT and MT running regarding the push-off forces during work-rates matched relative to the physiological thresholds to verify if this can explain the [Lac] differences at the iLT found here.

## Conclusions

This study's results show similar ${\dot{\text V}}$˙O_2max_ and ${\dot{\text V}}$˙O_2_ at iRCP between tethered running at NMT and conventional running at MT, but poor agreement for blood lactate concentrations at iLT, as determined by an incremental intensity test. Intensities associated with these thresholds were significantly correlated between NMT and MT, reinforcing concurrent validity. Therefore, running on an NMT offers higher glycolytic demand at the same relative internal load as the MT but with a similar aerobic response and correlated intensity determination (Supplementary Information).

## Supplementary Information

Supplementary Information.
