# Supplementary material for: Comparison of physiological responses of running on a nonmotorized and conventional motor-propelled treadmill at similar intensities
Source: Sci Rep. 2022 Jul 8;12:11626. doi: 10.1038/s41598-022-13741-w (PMC9270331; doi:10.1038/s41598-022-13741-w)
Supplement: Supplementary file 1 — Supplementary Information. [file 41598_2022_13741_MOESM1_ESM.docx]

Supplementary material

Considering the growing use of social media to enhance journals and manuscripts impact and reach to the interested public, we made a graphical abstract reuniting the major results of the paper. It can be used to help to spread key points quickly using twitter, Instagram or any other social media outlet.


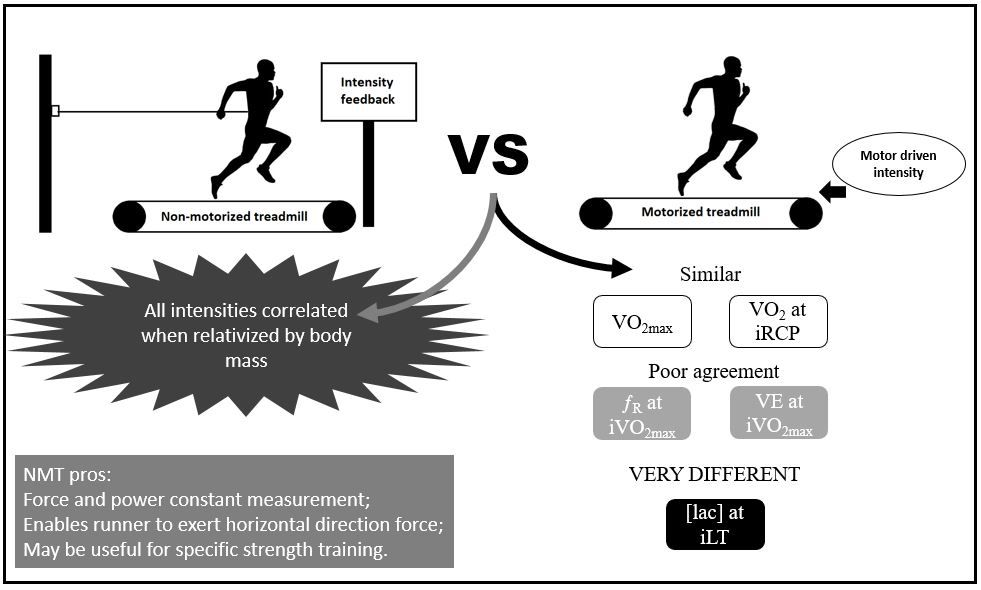


Figure label

**Supplementary Figure 1** – Graphical abstract reuniting key points of the paper, as well as the pros of using a NMT as an instrument to perform the classical incremental intensity test.
